# Comparative Chloroplast Genome Analyses Reveal a Fine-Scale Phylogenetic Framework and Cryptic Diversity in the *Fagopyrum dibotrys* Complex (Polygonaceae)

**DOI:** 10.3390/genes17020149

**Published:** 2026-01-28

**Authors:** Yi-Ming Wei, Xiao-Ting Xie, Shu-Qing Lei, Bo Li

**Affiliations:** 1 College of Agronomy, Jiangxi Agricultural University, Nanchang 330045, China; yimingwei2023@163.com; 2 Center for Integrative Conservation, Xishuangbanna Tropical Botanical Garden, Chinese Academy of Sciences, Mengla 666303, China; xiexiaoting@xtbg.ac.cn; 3 College of Life Sciences, University of Chinese Academy of Sciences, Beijing 100049, China

**Keywords:** cryptic diversity, geographic isolation, plastome evolution, sequence variation, fine-scale phylogeny, mutational hotspots, medicinal germplasm

## Abstract

**Background/Objectives:** The *Fagopyrum dibotrys* complex is a specialized high-altitude lineage in southwestern China with medicinal and breeding potential, but species delimitation remains unresolved. **Methods:** We sequenced 26 complete chloroplast genomes from the Hengduan Mountains to the Yunnan–Guizhou Plateau, analyzing genomic structures, variation patterns, and phylogenetic relationships. **Results:** All genomes exhibited typical quadripartite structures (152,213–160,302 bp), containing 133 genes (88 protein-coding, 8 rRNA, and 37 tRNA) with GC content of 37.9%. Collinearity analysis revealed highly conserved structures without structural rearrangements. Variations were concentrated in the large single-copy(LSC)/small single-copy(SSC) non-coding regions, with hotspots at *ycf4–cemA* and *ndhF–rpl32*. Codon usage showed an A/U bias, with leucine being most abundant and cysteine the least. Simple sequence repeats (SSRs) were predominantly mononucleotide repeats enriched in the LSC, while long repeats were mainly palindromic/forward. Maximum likelihood and Bayesian phylogenies consistently resolved three clades: Tibetan high-altitude specialists, limestone specialists, and a widespread Hengduan–Yunnan–Guizhou clade, with geographic clustering indicating isolation as the primary differentiation driver. **Conclusions:** This study refines the phylogenetic resolution of the *F. dibotrys* complex and identifies informative chloroplast markers, providing a genomic foundation for reliable species delimitation, evolutionary inference, and conservation management of this medicinal lineage.

## 1. Introduction

*Fagopyrum* (Polygonaceae) originated in the Hengduan Mountains–Himalayan region and radiated northward across Asia to Europe via the Silk Road, establishing a distribution pattern from East Asia to Europe [1,2,3,4,5,6]. As a specialized high-altitude taxon within this genus, the *F. dibotrys* complex is predominantly distributed in southwestern China at altitudes of 2000–4000 m, possessing significant medicinal value and breeding potential [7,8,9,10,11,12,13]. Specifically, its dried rhizomes are rich in flavonoids and other bioactive compounds, constituting the primary source of the traditional Chinese medicine “Jinqiaomai” [10,14]. Moreover, as wild relatives of cultivated buckwheat, species within this complex exhibit remarkable stress resistance in their native habitats, making them ideal genetic resources for breeding [15,16,17]. However, the realization of these application values is fundamentally constrained by persistent taxonomic ambiguity and unresolved interspecific relationships, which underscore the need for systematic genomic appraisal [8].

Classification of the genus *Fagopyrum* has evolved through deep integration of morphological and molecular evidence [15,18,19,20,21]. Ohnishi (1996) divided the genus into two functional groups based on seed size, isozyme profiles, and DNA polymorphism patterns—namely, the large-seeded *dibotrys* group and the small-seeded *Urophyllum* group—thereby establishing the core taxonomic framework for this complex [21]. Subsequent molecular systematic studies further revealed intricate interspecific relationships and potential hybridization history, reinforcing its “species complex” attribute [15,22,23]. Nevertheless, studies employing the internal transcribed spacer (ITS), *matK*, and *psbA–trnH* fragments, while effective at distinguishing the large- and small-seeded groups, offered limited resolution for elucidating relationships within the *F. dibotrys* complex [15,22,23,24]. Furthermore, although Li et al. (2022) identified two highly supported deep-level clades based on 20 complete plastomes, such coverage remains insufficient for detecting microgeographic differentiation [25]. At the broader scale, complete chloroplast genome sequences have increasingly become the preferred resource for resolving shallow-node phylogenies and cryptic species across angiosperms because they provide thousands of informative sites and reveal structural mutations invisible to fragment markers [26,27]. Recent reviews underscore that plastome phylogenomics now underpins maternal ancestry inference in crops such as *chrysanthemum* [28], and highlight the expanding role of organellar genomes in plant systematic and conservation research [29]. Within *Fagopyrum*, the pan-plastome strategy has markedly enhanced intraspecific phylogenetic resolution via large-scale sampling. For instance, Zhou et al. (2023) identified 7709 polymorphic sites from 513 *F. tataricum* plastomes, successfully delineating three main genetic clusters and their subpopulation structures [30]. By contrast, plastome-level sampling of the wild *F. dibotrys* complex remains sparse, constraining the power to detect microgeographic differentiation [25]. Moreover, nuclear-plastid topological conflicts detected in the *Urophyllum* group suggest that similar genetic signal heterogeneity may exist within the *F. dibotrys* complex, necessitating validation through high-resolution plastome data [6,15,23,25,31].

In recent years, the chloroplast genome, has become an ideal resource for plant phylogenetic studies [26,32,33]. Compared with traditional fragment data, complete plastome sequences can enhance phylogenetic resolution by increasing the number of informative sites, and their structural variations (such as inverted repeat (IR) region expansion/contraction and single-copy (SC)/IR boundary shifts) provide complementary information for species delimitation [27,34,35]. Although chloroplast genomes have proven valuable for genus-level phylogenetic studies in *Fagopyrum* [36], a pioneering study that reported the first complete chloroplast genome of *F. dibotrys*, the *F. dibotrys* complex has yet to receive systematic whole-plastome investigation, particularly regarding the reconstruction of high-resolution phylogenetic networks and the evaluation of structural variation in resolving interspecific relationships. Here, we aim to generate and analyze a comprehensive set of complete chloroplast genomes from the *F. dibotrys* complex to: (1) construct a high-resolution phylogenetic framework for resolving interspecific relationships, (2) identify plastome structural variations and mutation hotspots with potential species-diagnostic value, (3) assess microgeographic differentiation and lineage divergence mechanisms driven by geographic isolation, and (4) provide genomic resources to guide taxonomic revision, conservation, and utilization of this medicinally important germplasm.

## 2. Materials and Methods

### 2.1. Sampling, DNA Extraction and Sequencing

#### 2.1.1. Sample Collection

To ensure comprehensive sample diversity, a three-phase sampling strategy was employed to collect 26 accessions of the *F. dibotrys* complex. Wild samples were obtained during field expeditions in western Sichuan Province in September 2024. Additional collections were made at a buckwheat breeding base in Sichuan in October 2024. To further broaden the geographical representation, research materials were also sourced from existing herbarium specimens. All samples were sourced from natural habitats, with detailed metadata on geographic coordinates, ecological conditions, and plant phenology recorded at the time of collection. Sampling procedures strictly complied with local laws and regulations and adhered to ecological protection principles to ensure legal and sustainable collection. Immediately after collection, samples were stored in silica gel to preserve DNA integrity for subsequent extraction and sequencing.

#### 2.1.2. Genomic DNA Extraction and Quality Assessment

Genomic DNA (gDNA) was isolated from plant tissues using the Plant DNA Extraction Kit (Tiangen Biotech, Beijing, China). The quality and yield of the extracted DNA were evaluated using agarose gel electrophoresis to assess integrity and the Qubit fluorometer (Invitrogen, Thermo Fisher Scientific, Waltham, MA, USA) for precise quantification of yield. Only samples with a DNA concentration of at least 5 ng/μL and a total yield of at least 200 ng were deemed high-quality and chosen for further library construction.

#### 2.1.3. Whole-Genome Sequencing Library Construction and Sequencing

To construct whole-genome sequencing (WGS) libraries, the Hieff NGS OnePot Pro DNA Library Prep Kit (Yeasen Biotechnology, Shanghai, China) for Illumina was employed. The process began with enzymatic fragmentation of 200 ng of gDNA, followed by end repair and 3′ adenylation. Adapter ligation was then performed, and the ligated products were purified and size-selected using kit-provided magnetic beads. After amplification using kit-compatible indexed primers, the amplicons were further purified using kit-provided magnetic beads. Finally, sequencing was carried out on the DNBSEQ-T7 sequencer (MGI Tech, Shenzhen, China) using the paired-end 150 bp (PE150) mode to generate high-quality sequencing data.

### 2.2. Genome Assembly and Annotation

De novo assembly of the complete chloroplast genomes of the *F. dibotrys* complex was performed using GetOrganelle v1.7.7.0 (https://github.com/Kinggerm/GetOrganelle, accessed on 27 December 2024) [37,38,39,40,41]. Assembly graphs were visualized and validated using Bandage [41].

Annotation was conducted using PGA v1.2 (https://github.com/quxiaojian/PGA, accessed on 27 December 2024), [42]. A multi-species reference set comprising GenBank files of *Amborella trichopoda* (basal angiosperm outgroup), *Fagopyrum dibotrys* (target taxon), *Fagopyrum longistylum* (congeneric relative), and *Nicotiana otophora* (model species for functional validation) was used for homology-based annotation. The annotation was performed in Linux in circular genome mode. Manual curation was performed using Geneious Prime 2025.0.2 to correct potential errors and validate gene functional annotations [43].

Circular genome maps were generated using OGDRAW (https://chlorobox.mpimp-golm.mpg.de/OGDraw, accessed on 27 December 2024) [44].

### 2.3. Comparative Chloroplast Genome Analyses

To evaluate sequence divergence levels, whole chloroplast genome alignments were performed using the Shuffle-LAGAN model in mVISTA (https://genome.lbl.gov/vista/mvista/submit.shtml, accessed on 30 December 2024), with the *F. dibotrys* reference MH196562 downloaded from NCBI [45,46]. Synteny analysis was conducted using the Mauve plugin in Geneious Prime 2025.02 with the following parameters: match seed weight = 15, minimum LCB score = 30,000, ‘Calculate Locally Collinear Blocks’ and ‘Full alignment’ options enabled, and MUSCLE 3.6 used as the alignment tool for gapped alignment [43,47]. Nucleotide diversity (Pi) was calculated using DnaSP 6.0 (https://link.gitcode.com/i/49d54e495480510afd1d0665bd574c4b?uuid_tt_dd=10_35279577700-1735991415075-188502&isLogin=9&from_id=142974385, accessed on 27 January 2026) via the sliding window method (window length = 600 bp, step size = 200 bp), with sites containing alignment gaps excluded to identify mutation hotspots [48]. Contraction and expansion of the IR regions were visualized using IRscope v1 and CPJSdraw v1 [49,50].

### 2.4. Analysis of Repetitive Sequences

SSRs were identified using MISA v2.1 with parameters set as: mononucleotide repeat units ≥ 10, dinucleotide repeats ≥ 5, trinucleotide repeats ≥ 4, and tetra-/penta-/hexanucleotide repeats ≥ 3 [51]. Dispersed repeat sequences (including forward, reverse, palindromic, and complementary repeats) were detected using the REPuter web server with a minimum repeat length of 30 bp and Hamming distance of 3 [52].

### 2.5. Codon Usage Analysis

Protein-coding sequences (CDSs) > 300 bp, without redundancy, starting with ATG and ending with TAA, TAG, or TGA were extracted using a custom Python v3.12.2 script. Codon usage bias was assessed with CodonW v1.4.2, including Relative Synonymous Codon Usage (RSCU) analysis [53]. RSCU values were visualized using TBtools v2.363 [54].

### 2.6. Phylogenetic Analysis

To infer phylogenetic relationships within the *F. dibotrys* complex, 26 de novo-sequenced plastome sequences were analyzed. Sequences underwent terminal trimming and manual correction of IR orientations in Geneious Prime v2025.02. Multiple sequence alignment was performed using MAFFT v7.520 (--auto, 1000 iterations), followed by visual inspection of misalignments in non-coding regions [43,55]. Using jModelTest-2.1.10, the GTR + Gamma model was selected [56]. Maximum likelihood (ML) analysis was performed using RAxML-NG v1.2.0 with the GTR + G + FO substitution model, replicate searches from 20 parsimony and 20 random starting trees [57]. Bayesian inference (BI) was conducted using MrBayes v3.2.7, specifying the GTR + Gamma + I model (4 discrete categories), running 2 independent analyses each with 4 Markov chain Monte Carlo (MCMC) chains (1 cold chain + 3 heated chains, heating temperature default 0.2), until the average standard deviation of split frequencies (ASDSF) fell below 0.01 (maximum 20,000,000 generations), sampling every 1000 generations, discarding the first 25% as burn-in before constructing consensus trees and calculating posterior probabilities, with random seeds generated by default [58]. Finally, ML and BI trees were visualized in FigTree v1.4.4 (https://github.com/rambaut/figtree/releases/tag/v1.4.4, accessed on 2 January 2025), with node support thresholds ML ≥ 70% and BI ≥ 0.95 [59].

## 3. Results

### 3.1. Chloroplast Genome Characteristics of Fagopyrum dibotrys Complex

**Figure 1:**
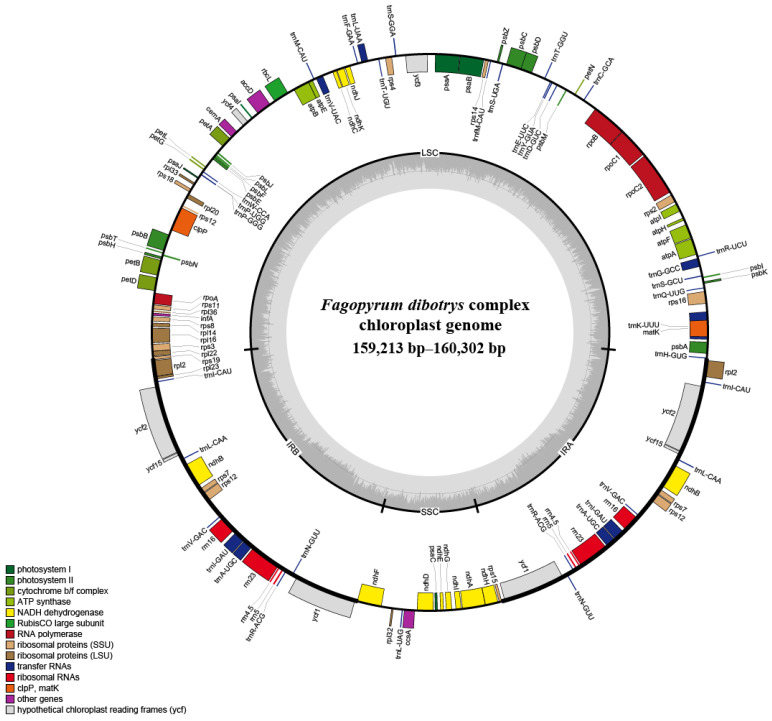
Complete plastome map of 26 samples of the *F. dibotrys* complex. The genes located outside of the circle are transcribed counterclockwise, while those located inside are transcribed clockwise.

The complete chloroplast genomes ranged from 152,213 bp (*w6*, *7*, *17*) to 160,302 bp (*w33*), with the LSC, SSS and IR regions spanning 84,374–85,359 bp, 13,241–13,292 bp, and 30,784–30,840 bp, respectively, and a consistent GC% content of 37.9%. Each plastome contained 133 genes, comprising 88 protein-coding genes, 8 rRNA genes, and 37 tRNA genes (Figure 1; Table S1). To characterize the genome composition of the *F. dibotrys* complex, plastid genes were annotated and grouped into three categories: photosynthesis, self-replication, and other genes (Table S2).

### 3.2. Comparative Genomic Analysis

**Figure 2:**
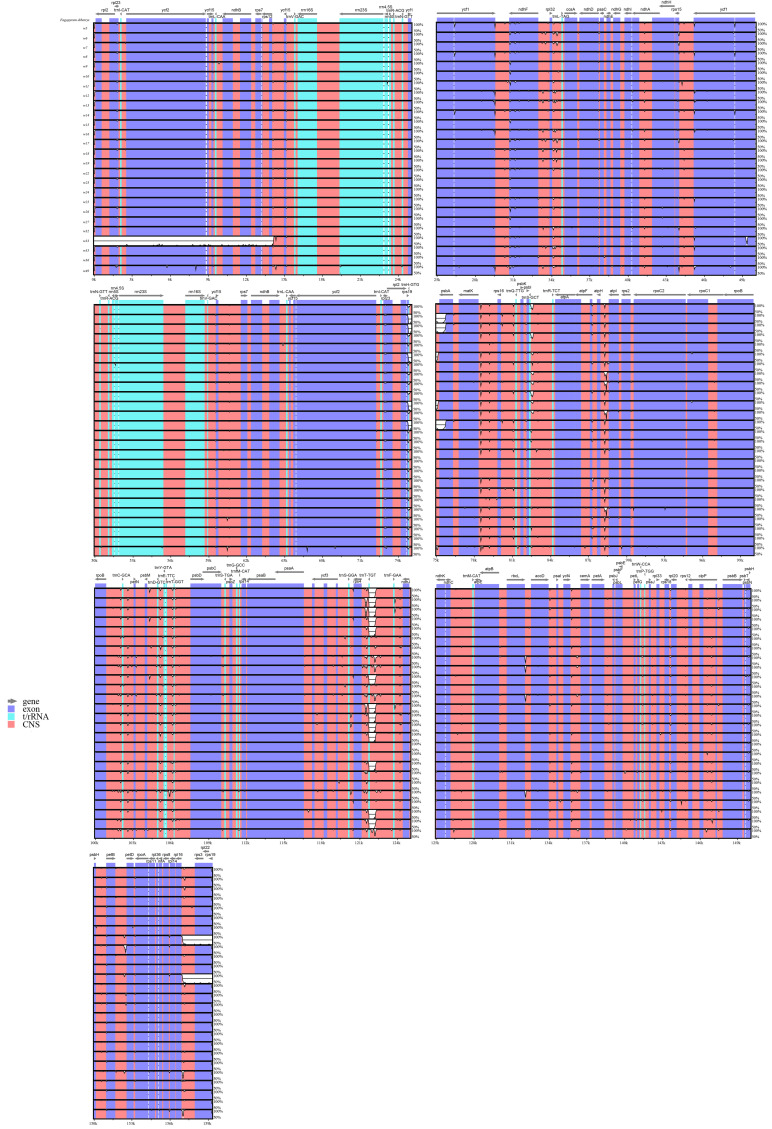
Percent identity plot comparing chloroplast genomes of the *F. dibotrys* complex using mVISTA.

The chloroplast genomes were largely conserved, with nucleotide differences detected only in the *psbA* gene region and five intergenic spacers: *trnH-GTG–psbA*, *atpI–atpH*, *trnT-TGT–rps4*, *trnF-GAA–trnT-TGT*, and *rbcL–accD*. Specifically, the *psbA* gene region and *trnT-TGT–rps4* intergenic spacer exhibited a consistent variation pattern in three samples (*w6*, *w7*, and *w17*), distinguishing them from the remaining samples. Similarly, the *atpI–atpH* and *rbcL–accD* intergenic spacers showed a shared variation pattern in four samples (*w12*, *w13*, *w16*, and *w32*), separating this subset from others (Figure 2). Overall, these results indicate that chloroplast genome variation in *F. dibotrys* complex primarily concentrated in intergenic regions, and the identified divergent intervals may serve as preliminary molecular markers for discriminating geographically distinct samples.

Syntenic analysis revealed that the chloroplast genomes of 26 samples exhibit highly conserved linear arrangements across all accessions. Locally Collinear Blocks (LCBs) were identical in length, order, and orientation with no evidence of structural rearrangement such as inversions, translocations, fragment deletions, or lineage-specific expansions (Figure S1). These results indicate that chloroplast genome structure in this complex is highly conserved.

Regarding IR expansion and contraction, variation was detected in the *ycf1* gene at the boundaries between the inverted repeat and single-copy regions, specifically at the Junction of SSC and inverted repeat B (JSB) and the Junction of SSC and inverted repeat A (JSA) (Figure S2). Within the IR regions, the *ycf1* gene ranged from 5700–5730 bp, with distances from the boundary varying between 265–288 bp. At the JSB boundary, the *ndhF* gene extended into the IRb region by 67–90 bp, while its length in the SSC region ranged from 2160–2189 bp. At the JSA boundary, the *rps15* gene was located in the SSC region with a constant length of 252 bp, although its distance from the JSA boundary varied from 2 bp to 23 bp. Additionally, the lengths of LSC, IR, and SSC regions differed among samples. For example, LSC region lengths ranged from 84,100 bp (e.g., *w5*, *w8*, *w14*) to 85,359 bp (*w33*), IR region from 30,784 bp (*w9*) and 30,840 bp (*w33*), and the SSC from 13,241 bp (*w32*) to 13,623 bp (*w33*). These variations in IR boundaries and genome fragment lengths indicate structural polymorphisms within the complex.

### 3.3. Codon Usage Bias and Amino Acids Frequency Analysis

**Figure 3:**
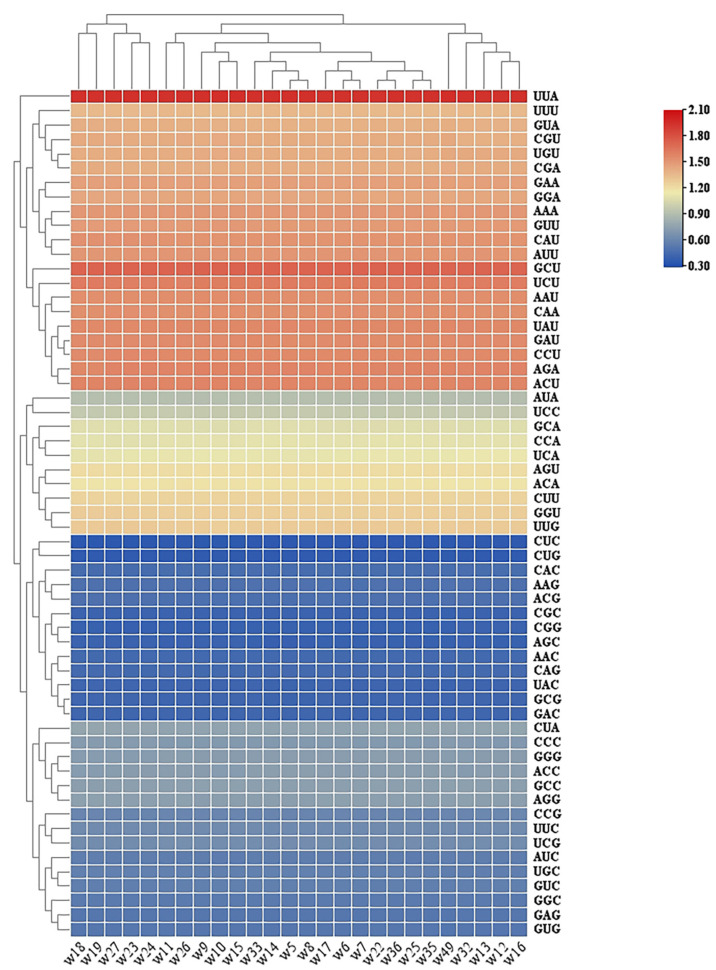
Heatmap of relative synonymous codon usage (RSCU) values among the 26 *F. dibotrys* complex samples.

Total number of codons in the chloroplast genomes of 26 samples from the *F. dibotrys* complex ranged from 16,635 (samples *w23*, *w24*, *w27*) to 16,891 (sample *w49*). Analysis of RSCU showed that 30 codons had RSCU values > 1 (accounting for 50.8%), of which 29 ended with A/U bases (96.7%), while 29 codons had RSCU values < 1 (49.2%), with 26 ending in G/C bases (89.7%), indicating an overall A/U bias across the chloroplast genomes (Figure 3).

At the amino acid level, leucine (Leu) was the most frequently used amino acid, with its synonymous codon UUA exhibited the highest RSCU value, whereas cysteine (Cys) was the least frequently used, with UGC showing the lowest RSCU value. The heatmap revealed distinct clustering of codons based on RSCU values: codons such as UUA, GCU, UCU, AGA, and ACU displayed strong usage preference (RSCU approaching or exceeding 1.5; represented in red/orange color), whereas CUC, CUG, CAC, ACG, and CGG showed weak usage preference (RSCU below 0.6; represented in blue color). This codon usage pattern likely reflects the combined effects of evolutionary forces and functional constraints in this taxonomic group (Figure 3).

### 3.4. Nucleotide Polymorphism Analysis

**Figure 4:**
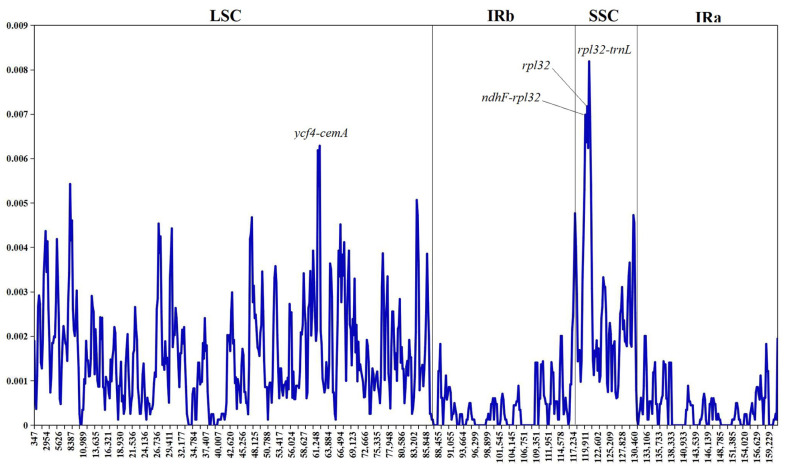
Sliding window analysis of the 26 whole chloroplast genomes of the *F. dibotrys* complex.

Sliding window analysis revealed that nucleotide diversity (Pi values) across the 26 chloroplast genomes ranged from 0 to 0.0082 with a mean of 0.0012, indicating low overall variation. Pi values were unevenly distributed across genomic regions: the two IR regions exhibited the lowest Pi values, reflecting their high sequence conservation, whereas the LSC and SSC regions contained distinct variation hotspots. Notably, Pi values exceeding 0.06 were observed at three intergenic loci–*ycf4–cemA* (LSC), *ndhF–rpl32* and *rpl32–trnL* (SSC) (Figure 4). These results demonstrate that sequence variation in the *F. dibotrys* complex primarily accumulates in specific non-coding regions of the single-copy regions, while IR regions and coding sequences remain highly conserved.

### 3.5. Repeat Sequence Analysis

**Figure 5:**
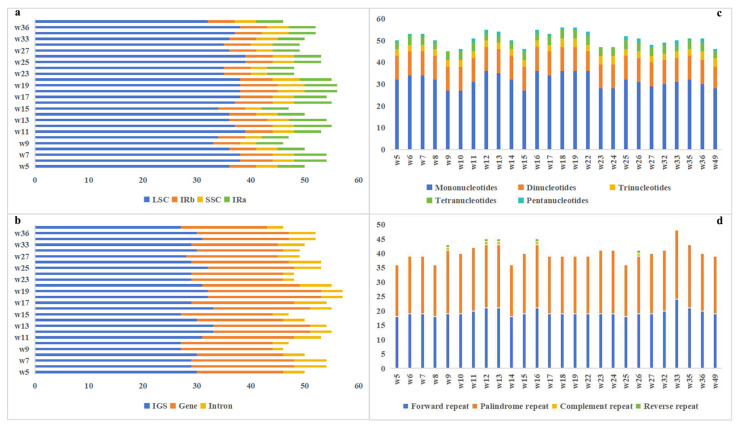
Identification of dispersed repeat sequences and SSRs in the chloroplast genomes of 26 *F. dibotrys* complex samples: (**a**) distribution of SSRs in the LSC, SSC, and IR regions. Red, yellow, and blue blocks represent the LSC, IR, and SSC regions, respectively; (**b**) distribution of SSRs in the IGS, Gene and Intron regions; (**c**) number of SSRs in the chloroplast genomes of these samples; (**d**) number of dispersed repeat sequences of different lengths in these chloroplast genomes.

The LSC region exhibited the highest abundance of SSR, the IR regions (inverted repeat A, inverted repeat B) had intermediate levels, and the SSC region showed the lowest abundance (Figure 5a). SSRs were further categorized into intergenic spacers (IGS), gene regions, and intron regions (Figure 5b), revealing that most SSRs were located in IGS regions, followed by gene regions, with intron regions containing the fewest. Additionally, SSRs were classified by motif length (mono-, di-, tri-, tetra-, and pentanucleotide repeats; Figure 5c), revealing that mononucleotide motifs were the most abundant, followed by dinucleotide motifs. Trinucleotide and tetranucleotide motifs showed similar abundances, while pentanucleotide motifs were the least common. These patterns indicate that SSRs predominantly occur in the LSC region and non-coding IGS regions, with short repeat motifs (mono- and dinucleotides) being dominant, consistent with evolutionary trends in plant chloroplast genomes.

For long sequence repeats (LSRs), we analyzed their types (forward, palindromic, complementary, and reverse repeats; Figure 5d). Palindromic and forward repeats were similarly abundant, with palindromic repeats slightly more frequent, whereas complementary and reverse repeats were rare and observed only in a few samples.

### 3.6. Phylogenetic Relationships

**Figure 6:**
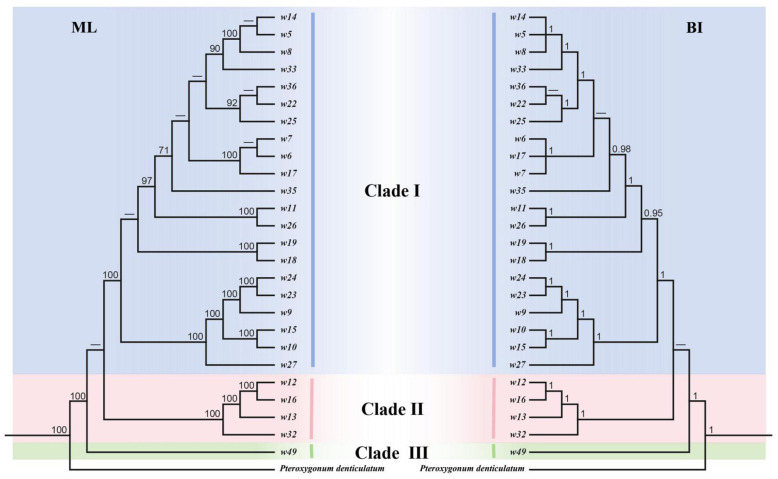
Phylogenetic relationships of 26 *F. dibotrys* complex samples. Left tree: maximum likelihood (ML); right tree: Bayesian inference (BI). Nodes with support values < 70% (ML)/0.95 (BI) are indicated by “–”.

**Figure 7:**
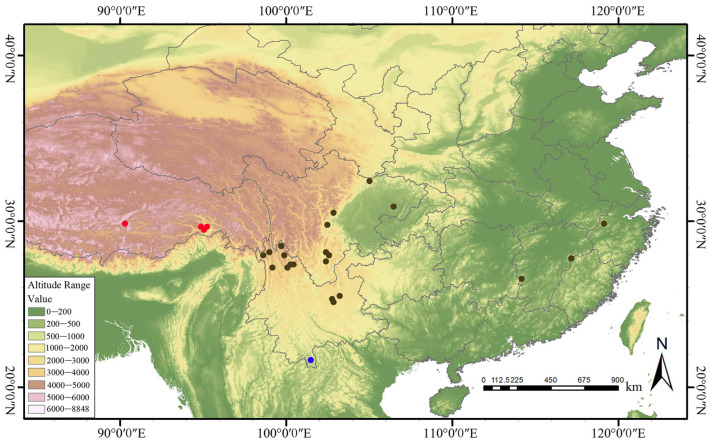
Geographic distribution of the 26 sampled accessions of the *F. dibotrys* complex. Symbols correspond to the three major phylogenetic clades identified in Figure 6: brown circles (Clade I), red circles (Clade II), and blue circles (Clade III).

Phylogenetic analysis resolved three major clades that show a clear geographic structure (Figure 6 and Figure 7): Clade III comprised a single operational taxonomic unit sample *w49* from limestone mountains, reflecting its unique phylogenetic position; Clade II included Tibetan samples (*w12*, *w16*, *w13*) forming a well-supported monophyletic group in both trees, with maximum bootstrap support of 100 in the ML tree and posterior probabilities ≥ 0.95 in the BI, demonstrating strong regional endemism; Clade I encompassed all remaining samples and was subdivided into subclades with clear geographical signals—for example, samples from the Yunnan-Guizhou Plateau (*w14*, *w5*), Hengduan Mountains (*w8*, *w6*, *w7*), Kunming (*w22*, *w25*), Lijiang (*w17*, *w19*, *w18*), Sichuan (*w26*, *w27*), and Yangtze River Basin (*w9*, *w10*, *w15*) each formed monophyletic subclades, reflecting significant genetic differentiation driven by geographical isolation. Notably, the clade comprising Hengduan Mountain samples *w6*, *w7* and Lijiang sample *w17* remained monophyletic in both trees. Minor topological differences between ML and BI trees likely arise from methodological characteristics rather than biological conflict. Partition-wise analysis of the concatenated protein-coding sequences yielded a topology highly similar to that of the whole-plastome matrix, with only minor rearrangements within terminal clades. Overall, these three major clades are well supported and strongly correlated with geographical distribution, validating the role of geography factors in shaping the phylogenetic structure of the *F. dibotrys* complex.

## 4. Discussion

### 4.1. Plastome Structural Diversity in Fagopyrum dibotrys Complex

The chloroplast genome is generally highly conserved, exhibiting a relatively low evolutionary rate, and serves as an important resource for plant molecular systematics and ecological studies [26,32,33,60,61,62,63]. This stability is particularly valuable for elucidating genetic relationships and evolutionary characteristics of medicinal plants, offering more precise phylogenetic insights [27,62,64]. Comparative analyses revealed that the chloroplast genomes of the *F. dibotrys* complex follow the conserved evolutionary patterns in angiosperms [65,66,67]: all samples retained the typical quadripartite structure, consistent with other *Fagopyrum* species (e.g., *F. tataricum*, *F. esculentum*) [25,30]; GC content was constant at 37.9%, gene composition and number (133 genes, including 88 protein-coding, 8 rRNA, and 37 tRNA genes) were highly conserved consistent with earlier studies, while collinearity analysis detected no rearrangement events, consistent with earlier studies [25,30,36]. These findings suggest the structural conservation and stability of the *F. dibotrys* complex genome.

### 4.2. Distribution Patterns of SSRs and Long Repeats

Simple sequence repeats (SSRs) in chloroplast genomes render them effective molecular markers for detecting population polymorphisms, while SSR polymorphism analysis also offers cost-effectiveness and high efficiency in phylogenetic studies of closely related species [68]. In this study, SSRs exhibited significantly higher abundance in the large single-copy (LSC) region compared to the inverted repeat (IR) and small single-copy (SSC) regions, predominantly enriched in intergenic spacers (IGS), with mononucleotide repeats representing the dominant type. This distribution pattern has been widely reported in chloroplast genomes of angiosperms [65,66,67]. Analysis of long sequence repeats (LSRs) demonstrated that palindromic and forward repeats predominated; however, most LSR variations were sporadically distributed, with reverse and complementary repeats observed in only a few individuals. Given the low distribution frequency of these rare repeat variants, their phylogenetic informativeness could not be fully evaluated under the current sample size. Referencing the requirement for large-scale sampling in the pan-plastome study of *F. tataricum*, future research should further expand population-level sampling density to systematically validate the practical application potential of these markers in microgeographic differentiation of the *F. dibotrys* complex [30].

### 4.3. Plastome-Based Phylogeny Supports Geography-Driven Differentiation in F. dibotrys Complex

Previous phylogenetic studies of the *F. dibotrys* complex have suffered from two major limitations: First, they relied primarily on fragmentary data such as ITS and matK [15,22,23,24], which contain sparse informative sites and thus insufficient resolution to elucidate internal relationships within this complex. Compared with fragment markers, the whole-genome approach—by covering more variable regions including non-coding areas—yielded phylogenetic signals supporting geographically structured clades (bootstrap support, BS ≥ 70; posterior probability, PP ≥ 0.95) in this study, with hotspots such as *ycf4–cemA* and *ndhF–rpl32* contributing key informative sites. Second, recent limited plastome studies have not achieved dense geographic coverage across its entire distribution range (from the Hengduan Mountains to the Yunnan–Guizhou Plateau) [25]. Consequently, the resolution of these studies was inadequate for detecting microgeographic differentiation signals that are crucial for understanding species delimitation in this medicinal group. Li et al. (2022) broadly split the *F. dibotrys* complex into two plastome lineages within sub-clade V, yet their inference rested on only three accessions with limited geographic coverage [25]. By expanding the sample set to 26 accessions covering the entire natural distribution range, the phylogenetic topology constructed in this study revealed a three-clade divergence pattern that is consistent with the geographic distribution of this complex: a limestone-habitat clade (Clade III), a Tibetan high-altitude clade (Clade II), and a widespread clade comprising samples from the Yunnan–Guizhou Plateau, Hengduan Mountains, and Yangtze River Basin (Clade I), reflecting genetic divergence driven by geographic isolation. This phylogenetic framework provides genomic evidence for the precise conservation and sustainable utilization of *F. dibotrys* complex germplasm resources based on geographic lineages. To understand the evolutionary forces underlying this structure, we synthesized previously published Ka/Ks estimates for *Fagopyrum* chloroplast genes. Collectively, these data reveal that the majority of loci exhibit ratios significantly below 1, indicative of pervasive purifying selection that accounts for the highly conserved plastome architecture documented in this study [25,36]. By contrast, two independent studies to date have consistently recovered Ka/Ks > 1 for *ndhK*, *petL*, *rpoC2*, *ycf1* and *ycf2*, implying episodic positive selection that may be associated with adaptation to high-elevation environments [25,36]. These previously reported signatures furnish candidate adaptive markers for future tests of whether the three geographic clades identified herein reflect ecologically driven divergence.

Despite these advances, the current sampling size remains insufficient for fully reconstructing the population history of this wild complex, which may harbor cryptic species [30]. Future studies should adopt a pan-plastome strategy with expanded population-level sampling density and integrate nuclear genomic data to thoroughly assess species boundaries and evolutionary processes in this important medicinal germplasm resource.

## 5. Conclusions

Our plastome analysis demonstrates that the *F. dibotrys* complex possesses a highly conserved chloroplast genome structure, with variation mainly confined to specific intergenic regions of the LSC and SSC. SSRs and long repeats were unevenly distributed, providing potential markers for detecting micro-level differentiation. Phylogenetic reconstruction resolved three well-supported geographically structured clades, supporting a geography-driven divergence model. Overall, this study establishes complete plastomes as powerful tools for clarifying evolutionary relationships and provides a genomic foundation for future population, evolutionary, and conservation research on the *F. dibotrys* complex.

## Data Availability

The data presented in this study are openly available in the National Center for Biotechnology Information (NCBI). During NCBI’s processing, the 26 samples were divided into two batches automatically. The first batch (1 sample, isolate *w5*) is accessible at [https://www.ncbi.nlm.nih.gov/nuccore/PX781602, accessed on 6 January 2026], with the accession number PX781602 (public release date: 5 January 2026). The second batch (25 samples) includes isolates *w6* to *w49*, with the accession numbers ranging from PX800879 to PX800903; each sample can be retrieved via the link format https://www.ncbi.nlm.nih.gov/nuccore/PX800879 (replace the serial number with the corresponding one in the range of PX800879-PX800903) (public release date: 10 January 2026). All data will be synchronized to the European Nucleotide Archive (ENA) and DNA Data Bank of Japan (DDBJ) after release.

## Supplementary Materials

The following supporting information can be downloaded at: https://www.mdpi.com/article/10.3390/genes17020149/s1, Figure S1. Collinear block-based analysis of 26 *Fagopyrum dibotrys* complex plastomes using Mauve alignment. Figure S2. Comparison of LSC, IR, and SSC border regions among 26 chloroplast genomes of the *Fagopyrum dibetrys* complex. Table S1. Comparative analyses on the basic features of the plastomes of twenty-six samples from *Fagopyrum dibotrys* Complex. Table S2. Genes in the plastomes of twenty-six samples from *Fagopyrum dibotrys* Complex.
