# Supplementary figures and images for: Comparative Chloroplast Genome Analyses Reveal a Fine-Scale Phylogenetic Framework and Cryptic Diversity in the Fagopyrum dibotrys Complex (Polygonaceae)
Source: Genes (Basel). 2026 Jan 28;17(2):149. doi: 10.3390/genes17020149 (PMC12941001; doi:10.3390/genes17020149)

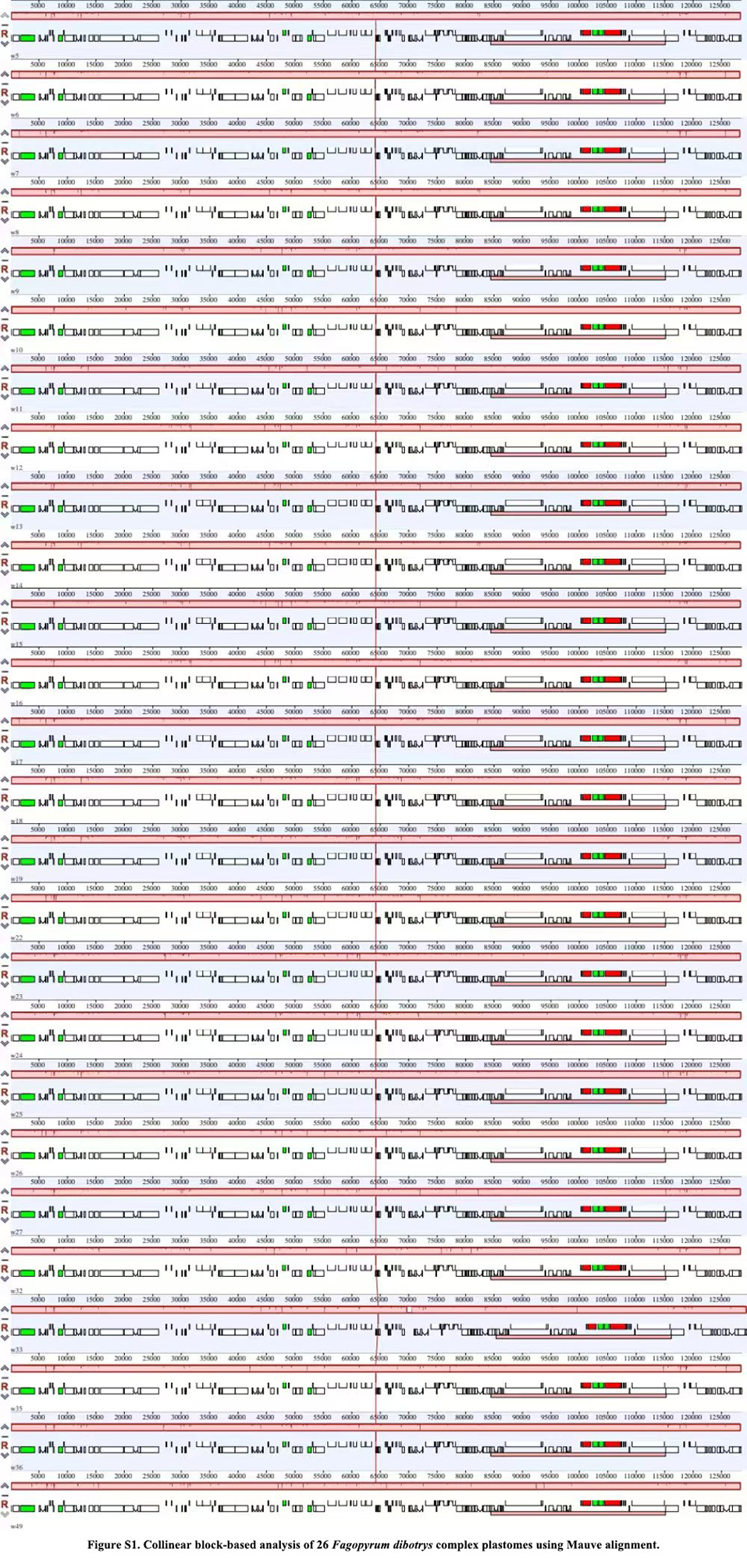

Supplement: Supplementary file 1 [file genes-17-00149-s001.zip › Figure S1. Collinear Mauve Fagopyrum dibotrys.png]
